# Supplementary material for: APOBEC3B regulates R-loops and promotes transcription-associated mutagenesis in cancer
Source: Nat Genet. 2023 Sep 21;55(10):1721–34. doi: 10.1038/s41588-023-01504-w (PMC10562255; doi:10.1038/s41588-023-01504-w)
Supplement: Supplementary file 1 — Supplementary Table 1 and Supplementary Note. [file 41588_2023_1504_MOESM1_ESM.pdf]

nature genetics

Article

https://doi.org/10.1038/s41588-023-01504-w

APOBEC3B regulates R-loops and promotes transcription-associated mutagenesis in cancer

In the format provided by the
authors and unedited

Supplementary information

Supplementary Table 1. Supporting data sets including sequence information

Layer 1 – Proteomic data from A3B and control AP-MS experiments

Layer 2 – NTS and TS mutations in >16-fold overexpressed gene groups

Layer 3 – Sequences of oligonucleotides

Supplementary Note

To analyze the kinetics of R-loop resolution in the presence and absence of *A3B*, global R-loop distributions were first analyzed by DRIP-seq in WT MCF10A following 2 hrs PMA treatment compared to DMSO control treatment (workflow in **Extended Data Fig. 4a**, left). As anticipated, PMA caused changes in the overall R-loop landscape with 13,422 peaks increased, 16,432 peaks decreased, and 171,322 unchanged (**Extended Data Fig. 4b-d**). Many genes such as *JUNB* and *FOS* were induced strongly by PMA treatment and showed significant increases in DRIP signals (**Extended Data Fig. 4e-f**). Other genes such as *NAXE* and *ARL4D* showed decreases in DRIP signals (**Extended Data Fig. 4g-h**), whereas the majority such as *GAPDH* and *GEMIN7* showed no changes in R-loop formation (**Extended Data Fig. 4i-j**). A parallel set of ChIP-seq experiments was done in MCF10A with Dox-inducible A3B-eGFP (**Fig. 1c**) to assess whether any of these R-loop categories might be bound by this deaminase (workflow in **Extended Data Fig. 4a**, right). An epitope-tagged protein was necessary because IP-grade antibodies have yet to be developed for endogenous A3B. Interestingly, A3B-eGFP appeared to bind preferentially to genomic DNA regions coincident with DRIP-seq peaks increased in PMA-

treated cells in comparison to vehicle control treated cells (ChIP-seq results superimposed over DRIP-seq results in **Extended Data Fig. 4b**). In contrast, A3B-eGFP ChIP-seq peaks were mainly unperturbed in genomic DNA regions in which R-loop levels decreased or remained unchanged upon PMA treatment (**Extended Data Fig. 4c-d**). Representative results were confirmed independently by ChIP-qPCR (**Extended Data Fig. 4k**). Moreover, quantification indicated that 43% of A3B ChIP peaks overlap with R-loop peaks induced by PMA (P = 0.0001, two-tailed binomial test; 54% overlap for ChIP peaks with >5-fold enrichment compared to uninduced Dox^- condition, P = 0.0008). These results indicate that A3B binds preferentially to genomic DNA regions with evidence for R-loop accumulation.

The PMA-inducibility of this system enabled an assessment of the kinetics of R-loop resolution in the presence and absence of A3B. WT and KO cells were treated with PMA or DMSO for 2 and 6 hrs and then analyzed by DRIP-seq (workflow in **Fig. 6a**). These timepoints were chosen for analysis because transcription induction and R-loop formation are rapid (2 hrs data above) and A3B protein levels are upregulated maximally by 6 hrs post-PMA treatment[30] (**Fig. 6b-c**). For example, *JUNB* and *DUSP1* have R-loop peaks that are strongly induced by PMA at 2 hrs and these return to near-control levels by 6 hrs (**Fig. 6d**). In contrast, R-loop peaks in the same genes remained significantly elevated and/or showed delayed resolution kinetics in KO cells after 6 hrs PMA treatment (**Fig. 6d**). PMA non-responsive control genes did not show R-loop induction or major differences in R-loop levels after 6 hrs PMA treatment (*e.g.*, *GAPDH* and *HSPA8* in **Fig. 6e**). Moreover, independent IF confocal microscopy studies of the kinetics of R-loop resolution following PMA treatment of WT MCF10A cells showed that nucleoplasmic R-

loop levels peak at 2 hrs and decline substantially by 6 hrs, whereas no decline (even a modest increase) was observed in *A3B* KO cells (**Fig. 6f-g**). As above, all DRIP-qPCR and nucleoplasmic R-loop signals were sensitive to RNase H treatment indicating specificity (**Fig. 6d-g**). Taken together with data from ChIP experiments, the results of these transcriptional activation experiments indicated that nuclear A3B is recruited to PMA-induced R-loops and contributes to their timely resolution.
