# Supplementary material for: APOBEC3B regulates R-loops and promotes transcription-associated mutagenesis in cancer
Source: Nat Genet. 2023 Sep 21;55(10):1721–34. doi: 10.1038/s41588-023-01504-w (PMC10562255; doi:10.1038/s41588-023-01504-w)
Supplement: Supplementary file 2 — Reporting Summary [file 41588_2023_1504_MOESM2_ESM.pdf]

Corresponding author(s): Reuben Harris
Last updated by author(s): Jul 17, 2023

# Reporting Summary

Nature Research wishes to improve the reproducibility of the work that we publish. This form provides structure for consistency and transparency in reporting. For further information on Nature Research policies, see our Editorial Policies and the Editorial Policy Checklist.

## Statistics

For all statistical analyses, confirm that the following items are present in the figure legend, table legend, main text, or Methods section.

| n/a | Confirmed | |
|---|---|---|
| ☐ | ☒ | The exact sample size (*n*) for each experimental group/condition, given as a discrete number and unit of measurement |
| ☐ | ☒ | A statement on whether measurements were taken from distinct samples or whether the same sample was measured repeatedly |
| ☐ | ☒ | The statistical test(s) used AND whether they are one- or two-sided<br>*Only common tests should be described solely by name; describe more complex techniques in the Methods section.* |
| ☐ | ☒ | A description of all covariates tested |
| ☐ | ☒ | A description of any assumptions or corrections, such as tests of normality and adjustment for multiple comparisons |
| ☐ | ☒ | A full description of the statistical parameters including central tendency (e.g. means) or other basic estimates (e.g. regression coefficient) AND variation (e.g. standard deviation) or associated estimates of uncertainty (e.g. confidence intervals) |
| ☐ | ☒ | For null hypothesis testing, the test statistic (e.g. *F*, *t*, *r*) with confidence intervals, effect sizes, degrees of freedom and *P* value noted<br>*Give P values as exact values whenever suitable.* |
| ☒ | ☐ | For Bayesian analysis, information on the choice of priors and Markov chain Monte Carlo settings |
| ☒ | ☐ | For hierarchical and complex designs, identification of the appropriate level for tests and full reporting of outcomes |
| ☒ | ☐ | Estimates of effect sizes (e.g. Cohen's *d*, Pearson's *r*), indicating how they were calculated |

*Our web collection on statistics for biologists contains articles on many of the points above.*

## Software and code

Policy information about availability of computer code

| | |
|---|---|
| Data collection | ImageJ (v1.48) NIH https://imagej.nih.gov/ij/<br>FV-10-ASW3.1 Olympus https://www.olympus-ims.com/en/<br>GraphPad Prism 6 N/A http://www.graphpad.com<br>Image Studio Li-COR Biosciences https://www.licor.com/bio/image-studio/<br>Typhoon FLA-7000 Image Reader GE Life Sciences<br>Li-COR Odyssey Fc Li-COR<br>NovaSeq 6000<br>Fluoview 3000 Confocal Olympus<br>Nikon AR1<br>BDLSRFortessa<br>Rotor Gene Q Thermocycler (QIAGEN) software v2.3.1 |

| | |
|---|---|
| Data analysis | Image J, FV-10-ASW3.1 Olympus and Fluoview 3000 Confocal Olympus and Nikon AR1were used for collection and quantification of immunofluorescence. GraphPad Prism v6, 8.0 and 8.3.1 was used for data plotting and statistical calculations. Image Studio Li-COR Biosciences was used for western blotting and dot blot quantifications. Cutadapt 1.13 (N/A https://cutadapt.readthedocs.io/en/stable/index.html), STAR 2.6.1d (N/A https://github.com/alexdobin/STAR), SAMtools 1.3.1 (N/A http://www.htslib.org/), Picard tools (N/A https://broadinstitute.github.io/picard/), Deeptools 2.5.0.1 (N/A https://deeptools.readthedocs.io/en/latest/index.html), MACS2 2.1.1.20160309 (N/A https://github.com/taoliu/MACS) and Bedtools version 2.29.2 were used for ChIP-seq and DRIP-seq data processing. Salmon 0.13.1 (N/A https://salmon.readthedocs.io/en/latest/index.html) was used for RNA-seq differential expression analysis. Typhoon FLA-7000 Image Reader GE Life Sciences and Li-COR Odyssey Fc Li-COR were used for western blot and dot blot imaging. Real-time quantitative PCRs (qPCRs) were analysed with the software v2.3.1 of Rotor Gene Q Thermocycler (QIAGEN). Li-COR Odyssey and Odyssey software were used for EMSA and A3B activity assay imaging and quantification. FlowJo was used for analysis of flow cytometry data (PI and EdU staining). |

For manuscripts utilizing custom algorithms or software that are central to the research but not yet described in published literature, software must be made available to editors and reviewers. We strongly encourage code deposition in a community repository (e.g. GitHub). See the Nature Research guidelines for submitting code & software for further information.

## Data

Policy information about availability of data

All manuscripts must include a data availability statement. This statement should provide the following information, where applicable:

- Accession codes, unique identifiers, or web links for publicly available datasets
- A list of figures that have associated raw data
- A description of any restrictions on data availability

The accession number for the ChIP-seq and DRIP-seq reported in this paper (Fig 5-6, S3 and S4) is GEO: GSE148581. The GEO accession number for the ChIP-seq, and DRIP-seq reported in this paper is: GSE148581. Protein-coding and non-coding genes annotation was taken from Gencode V31 and enhancers from FANTOM5, both based on hg38 version of the human genome. ChIP-seq and DRIP-seq peak calling files are available in the GEO submission GSE148581.

# Field-specific reporting

Please select the one below that is the best fit for your research. If you are not sure, read the appropriate sections before making your selection.

☒ Life sciences ☐ Behavioural & social sciences ☐ Ecological, evolutionary & environmental sciences

For a reference copy of the document with all sections, see nature.com/documents/nr-reporting-summary-flat.pdf

# Life sciences study design

All studies must disclose on these points even when the disclosure is negative.

| | |
|---|---|
| Sample size | No calculation or statistics were used to determine sample size. Sample size was determined according to established practice and applicable standard at affordable costs. Samples sizes are indicated separately for different experiments. Statistical analysis was used to determine statistical significance of obtained results (as indicated in figure legends). P-values are reported in figures and/or figure legends. |
| Data exclusions | No samples were excluded. |
| Replication | The exact sample size (n) for each experimental group/condition are provided in the figure legends. |
| Randomization | Randomization was not feasible for this type of molecular biology, biochemistry and genomics experiments. The culture cell plates were randomly assigned to each group for respective treatment and samples in the same experiments were treated in the same manner. |
| Blinding | Blinding was not possible for our experiments as data collection and analysis were performed by the same person. |

# Reporting for specific materials, systems and methods

We require information from authors about some types of materials, experimental systems and methods used in many studies. Here, indicate whether each material, system or method listed is relevant to your study. If you are not sure if a list item applies to your research, read the appropriate section before selecting a response.

## Materials & experimental systems

| n/a | Involved in the study |
|---|---|
| ☐ | ☒ Antibodies |
| ☐ | ☒ Eukaryotic cell lines |
| ☒ | ☐ Palaeontology and archaeology |
| ☒ | ☐ Animals and other organisms |
| ☒ | ☐ Human research participants |
| ☒ | ☐ Clinical data |
| ☒ | ☐ Dual use research of concern |

## Methods

| n/a | Involved in the study |
|---|---|
| ☐ | ☒ ChIP-seq |
| ☐ | ☐ Flow cytometry |
| ☒ | ☐ MRI-based neuroimaging |

# Antibodies

Antibodies used

Primary antibodies used in these experiments were:
Tubulin (Abcam, Cat# ab4074, RRID:AB_2288001, Clone EPR13478(B)). WB 1:5000
Tubulin (Abcam, Cat# ab6046, RRID:AB_2210370, lot#GR77827-1). WB 1:5000
Tubulin (Sigma-Aldrich Cat# T5168, RRID:AB_477579, lot#039M4769V). WB 1:10000
Flag (Sigma-Aldrich Cat# F1804, RRID:AB_262044, lot#SLCF9337). WB 1:10000
A3B (5210-87-13, inhouse13). WB 1:2000
Topoisomerase I (Abcam, Cat# ab109374, RRID:AB_10861978, clone EPR5375, lot #GR49853-20) WB 1:1000 or 1:500.
Lamin B1 (Abcam Cat# ab16048, RRID:AB_443298). WB 1:2000
anti-IgG2a (Sigma-Aldrich Cat# M5409, RRID:AB_1163691)
anti-HA (Cell Signaling Technology Cat# 3724, RRID:AB_1549585). WB: 1:5000
HNRNPUL1 (gift from Prof. Stuart Wilson, University of Sheffield, UK). WB 1:1000.
GFP (Abcam Cat# ab290, RRID:AB_303395, lot# GR3251545 and GR3270983). WB 1:3000. Co-IP 2.5 µg. ChIP 2.5 µg.
mCherry (Abcam Cat# ab167453, RRID:AB_2571870, lot# GR3209879-3). WB 1:1000.
IgG Isotype Control (Thermo Fisher Scientific Cat# 02-6102, RRID:AB_2532938, lot#RI238244). Co-IP 2.5 µg.
RNA/DNA hybrids clone S9.6 (Gromak Lab, University of Oxford Cat# Gromak_1, RRID:AB_2810829). Slot Blot 1:1000. RNA/DNA hybrid and protein co-immunoprecipitation 100 µl. DRIP 30 µl.
RNA/DNA hybrids clone S9.6 (Kerafast Cat# ENH001, RRID:AB_2687463, lot# 032119_2 and 032119_4). IF 1:200. Dot blot 1:200000
dsDNA (Abcam Cat# ab27156, RRID:AB_470907) Dot blot 1:300000
gamma-H2AX (Novus Cat# NB100-384, RRID:AB_10002815, lot# A22). IF 1:1000

Secondary antibodies used were:
Rabbit IRdye 800CW (LI-COR Biosciences Cat# 827-08365, RRID:AB_10796098) WB 1:10000
Mouse IRdye 680LT (LI-COR Biosciences Cat# 925-68020, RRID:AB_2687826) WB 1:10000
Rabbit HRP (Cell Signaling Technology Cat# 7074, RRID:AB_2099233) WB 1:10000
Rabbit IgG (whole molecule)-Peroxidase antibody produced in goat (Sigma-Aldrich Cat# A0545, RRID:AB_257896). WB 1:8000.
Mouse HRP (Cell Signaling Technology Cat# 7076, RRID:AB_330924) WB 1:10000
Mouse IgG (whole molecule)-Peroxidase antibody produced in goat (Sigma-Aldrich Cat# A8924, RRID:AB_258426). Slot Blot 1:3333.
Alexa Fluor 488 goat anti-mouse IgG (Molecular Probes Cat# A-11029, RRID:AB_2534088). IF 1:1000
Alexa Fluor 594 goat anti-mouse IgG (Molecular Probes Cat# A-11032, RRID:AB_2534091). IF 1:1000
Alexa Fluor 488 goat anti-rabbit IgG (Thermo Fisher Scientific Cat# A-11034, RRID:AB_2576217) IF 1:1000
Alexa Fluor 594 goat anti-rabbit IgG (Thermo Fisher Scientific Cat# A-11037, RRID:AB_2534095) IF 1:1000
Alexa Fluor 647 goat anti-mouse IgG (Thermo Fisher Scientific Cat# A-21236, RRID:AB_2535805). IF 1:1000

Validation

Rabbit polyclonal alpha Tubulin (ab4074) was validated by Abcam using WB (1 µg/ml dilution) in several human (including HeLa, NIH 3T3 and PC12) and mouse whole cell extracts. The specific WB band can be prevented by incubation with human alpha-tubulin peptide. https://www.abcam.com/alpha-tubulin-antibody-loading-control-ab4074.html.

Rabbit IgG Isotype control (02-6102) was used in literature as IP control (e.g. Ge Zhy, Mol Cell, 2019 PMID: 30846317; Cristini et al, Nat Commun, 2022 PMID:35618715). We further showed the absence of protein binding tested by WB following co-IP with this antibody (Fig 1d).

Rabbit polyclonal Lamin B1 antibody was validated by Abcam using WB (0.1-1 µg/ml dilution) in HeLa and A431 whole cell extracts. No band was detected upon LMNB1 (lamin B1) knock-out in HAP1 cells. https://www.abcam.com/lamin-b1-antibody-nuclear-envelope-marker-ab16048.html.

Rabbit monoclonal (EPR5675) anti-Topoisomerase I (ab109374) antibody was validated by Abcam using WB (1/10000 dilution) in several human whole cell lysates (e.g. MCF7, Jurkat, HepG2, K562). https://www.abcam.com/products/primary-antibodies/topoisomerase-i-antibody-epr5375-ab109374.html. Authors have previously validated the specificity of this ab for WB applications by showing a decreased band at 90 KDa in presence of siRNA targeting TOP1 in human U2OS and WI38 lysates (Mouly et al, Cell Death Dis, 2018 PMID: 30209297) and for usage in RNA/DNA and protein co-IP (Cristini et al, Cell Rep, 2018 PMID: 29742442; Abakir et al, Nat Genet, 2020 PMID: 31844323).

Mouse monoclonal RNA/DNA hybrid clone S9.6 antibody used in this study was previously validated for DRIP application by showing that RNase H digestion significantly removed S9.6 signal, indicating its specificity for RNA/DNA hybrids (Groh et al, PLoS Genet, 2014 PMID: 24787137; Cristini et al, Cell Rep, 2018 PMID:29742442; Cristini et al, Cell Rep, 2019 PMID: 31533039; Cristini et al, Nat Commun, 2022 PMID:35618715). We further validated the specificity of this antibody in our DRIP-qPCR experiments by using RNase H digestion in vitro control (Fig. 5g,i,k and 6d,e). S9.6 antibody was validated in Immunofluorescence experiments by expression of RNaseH, which processes R-loops and removes the specific RNA/DNA hybrid signal (see Kim et., 2019, Genes Dev, PMID: 31753913;

Bayona-Feliu et al., 2021, Nature Genetics, PMID: 33986538; Abakir et al., 2020, Nature Genetics 2020, PMID:31844323; Jurga M et al, 2021, Nat Commun, PMID: 34526504; Ramachandran et al, 2021, Nat Commun, PMID: 34140498; Pérez-Calero C et al, 2020, Genes Dev, PMID: 32439635).

Rabbit polyclonal anti-gamma H2AX (NB100-384) was validated by Abcam using a genetic strategy using Western blotting with extracts from H2AX WT cells and H2AX KO cells (from human HEK293, human melanoma (G361), mouse wildtype embryonic fibroblasts (+/+) or mouse H2AX knockout embryonic fibroblasts (-/-), either untreated or treated with the Neocarzinostatin, a DNA damaging agent and probed with antibody at 0.1 ug/ml.

Rabbit polyclonal anti-GFP (ab290) antibody was validated by Abcam using WB to detect the GFP fraction from cell extracts expressing recombinant GFP fusion proteins, notably in COS7 and LNCaP whole cell lysate transfected with GFP-Eml4. It is routinely used in immunoprecipitation and it has been validated in IP in HEK293 nuclear lysate expressing GFP versus lysates from cells with no GFP. https://www.abcam.com/products/primary-anti4bodies/gfp-antibody-ab290.html. It is also used for ChIP applications (https://www.abcam.com/content/anti-gfp) in human cells (Mei Tan-Wong et al, Mol Cell, 2019 PMID: 31679819).

Rabbit polyclonal anti-mCherry (ab167453) antibody was validated by Abcam using WB to detect the mCherry fraction from cell extracts expressing recombinant mCherry fusion proteins in HEK293 whole cell lysate transfected with pFin-EF1-mCherry vector. IF validation was performed similarly by Immunofluorescent analysis of HEK293 cells transfected with pFin-EF1-mCherry vector labeling mCherry with ab167453 at 1/500 dilution.

Rabbit polyclonal anti-hNRNPUL1 used in this study was a gift from Prof. Stuart Wilson, University of Sheffield, UK. The specificity of this antibody has been validated in the Gromak laboratory for WB applications by showing the decreased band at around 100 KDa in presence of siRNA targeting hNRNPUL1 in human cell lysates. This antibody has further been validated by IP.

Validation for anti-A3B (5210-87-13, in house) is published in Brown, W.L. et al. A rabbit monoclonal antibody against the antiviral and cancer genomic DNA mutating enzyme APOBEC3B. Antibodies (Basel) 8(2019).

# Eukaryotic cell lines

Policy information about cell lines

Cell line source(s)

U2OS cells were obtained from ATCC (ATCC HTB-96).
U2OS shCtrl and shA3B cell lines were made from U2OS cells (ATCC HTB-96) using previously described shCtrl and shA3B lentiviral constructs, viral production and transduction methods and puromycin selection 1 µg/mL1. U2OS pcDNA3.1-A3-3xHA stable lines were made via linear (NruI digested) transfection and selection using 800 µg/mL G418.
HEK 293T cells were obtained from ATCC (#CRL-3216).
MCF10A cells were obtained from ATCC (ATCC CRL-10317)
MCF10A-TREx-A3B-eGFP were maintained in the same MCF10A media described above with the addition of 100 µg/mL Normocin.
S9.6 Hybridoma cells were obtained from ATCC (ATCC HB-8730).
MCF10A A3B KO cell line was engineered by transduction of MCF10A cells (ATCC CRL-10317) with pLentiCRISPR, expressing the gRNA sequence GCTCCATTCAACCCCCCTGCT targeting both the A3A and A3B genes. Cells were selected with puromycin and seeded for single cell cloning. Deletion mutant lines were identified by PCR using primers amplifying unique sequences within the A3B gene and/or the A3A/B junction (primers in ref.2) and confirmed by qPCR and immunoblots.
HeLa cells were obtained from Nicholas Proudfoot (University of Oxford, UK) and are originally from ATCC.
U2OS A3B KO cell line was engineered from U2OS cells (ATCC HTB-96) by transduction with pLentiCRISPR, expressing the gRNA sequence GCGTGACGATCATGGACTAT targeting exon 3 of A3B. Cells were selected with puromycin and seeded for single cell cloning. Biallelic A3B knockout was confirmed by PCR using primers spanning the gRNA target region and subsequent sequencing in addition immunoblotting.

Authentication

STR profiling is used to authenticate the cell lines that were purchased from ATCC.
U2OS shCtrl and shA3B cell lines were validated using RT-qPCR and Western Blot for knockdown efficiency.
MCF10A-TREx-A3B-eGFP was validated using IF, Western blotting and RT-qPCR for inducible expression of A3B-eGFP.
MCF10A A3B KO was validated by PCR using primers amplifying unique sequences within the A3B gene and/or the A3A/B junction (primers in ref.2) and confirmed by qPCR and immunoblots.
U2OS A3B KO cell line was confirmed by PCR using primers spanning the gRNA target region and subsequent sequencing in addition immunoblotting.

Mycoplasma contamination

All cell lines were confirmed mycoplasma negative. MCF10A-TREx-A3B-eGFP had been cured of mycoplasma prior to initial studies.

Commonly misidentified lines
(See ICLAC register)

No commonly misidentified lines were used.

# ChIP-seq

## Data deposition

☒ Confirm that both raw and final processed data have been deposited in a public database such as GEO.

☒ Confirm that you have deposited or provided access to graph files (e.g. BED files) for the called peaks.

| | |
|---|---|
| Data access links<br>*May remain private before publication.* | The accession number for the ChIP-seq and DRIP-seq reported in this paper is GEO: GSE148581. |
| Files in database submission | ChIP-seq: Input and GFP IP -DOX, Input and GFP IP +DOX, Input and GFP IP +DOX +PMA in MCF10A-TREx-A3B-eGFP. DRIP-seq: Input and IP DMSO, PMA 2h, PMA 6h in MCF10A A3B WT or KO. |
| Genome browser session<br>(e.g. UCSC) | https://genome.ucsc.edu/s/TMichael2/082021Submission_APOBEC_ChIPseq_DRIPseq_hg38 |

## Methodology

| | |
|---|---|
| Replicates | 1. DRIP-seq and ChIP-seq results were validated by DRIP- and ChIP-qPCR. |
| Sequencing depth | For ChIP-seq: between 67 and 89 million unique and properly mapped paired-end reads per sample. For DRIP-seq: between 50 and 149 million unique and properly mapped paired-end reads per sample. Length of the reads: 150 bp. |
| Antibodies | anti-GFP (Abcam, ab290, lot# GR3251545 and GR3270983) for ChIP-seq and RNA/DNA hybrids (S9.6, Natalia Gromak Lab, University of Oxford Cat# Gromak_1, RRID:AB_2810829) for DRIP-seq. |
| Peak calling parameters | Adapters were trimmed with Cutadapt version 1.13 in paired-end mode with the following parameters: -q 15, 10 –minimum-length 10 -A AGATCGGAAGAGCGTCGTGTAGGGAAAGAGTGT -a AGATCGGAAGAGCACACGTCTGAACTCCAGTCA. Obtained sequences were mapped to the human hg38 reference genome with STAR version 2.6.1d and the parameters --runThreadN 16 --readFilesCommand gunzip -c –k --alignIntronMax 1 --limitBAMsortRAM 20000000000 --outSAMtype BAM SortedByCoordinate. Properly paired and mapped reads (-f 3) were retained with SAMtools version 1.3.1. PCR duplicates were removed with Picard MarkDuplicates tool. Reads mapping to the DAC Exclusion List Regions (accession: ENCSR636HFF) were removed with Bedtools version 2.29.2. FPKM-normalized bigwig files were created with deepTools version 2.5.0.1 bamCoverage tool with the parameters –bs 10 –p max -e --normalizeUsing RPKM.<br>ChIP-seq and DRIPseq peaks were called with MACS2 version 2.2.6. (Zhang et al., 2008) and the parameters: callpeak -f BAMPE -g 2.9e9 -q 0.01 --bdg --call-summits --nomodel --extsize 200. Each IP and its respective input were used as treatment and control, respectively. DRIP-seq differential peak calling was performed with MACS2 bdgdiff tool. The lists of called peaks are presented as the summit -/+ 250 bp (based on the --call-summits option of MACS2). |
| Data quality | Number of peaks with a fold enrichment > 5 and a q-value < 0.01 for ChIP-seq: 1,301 peaks for GFP IP DOX- DMSO, 610 peaks for GFP IP DOX+ DMSO, and 771 peaks for GFP IP DOX+ PMA 2h.<br>Number of peaks with a fold enrichment > 5 and a q-value < 0.01 for DRIP-seq: 30,920 peaks for WT DMSO, 48,537 peaks for WT +PMA 2h, 89,578 peaks for WT+PMA 6h, 33,794 peaks for KO DMSO, 60,404 peaks for KO+PMA 2h, 73,070 peaks for KO+PMA 6h. |
| Software | Adapters were trimmed with Cutadapt version 1.13 in paired-end mode. Obtained sequences were mapped to the human hg38 reference genome with STAR version 2.6.1d a. Properly paired and mapped reads (-f 3) were retained with SAMtools version 1.3.1. PCR duplicates were removed with Picard MarkDuplicates tool. eads mapping to the DAC Exclusion List Regions (accession: ENCSR636HFF) were removed with Bedtools version 2.29.2. FPKM-normalized bigwig files were created with deepTools version 2.5.0.1 b. |

# Flow Cytometry

## Plots

Confirm that:

☒ The axis labels state the marker and fluorochrome used (e.g. CD4-FITC).

☒ The axis scales are clearly visible. Include numbers along axes only for bottom left plot of group (a 'group' is an analysis of identical markers).

☒ All plots are contour plots with outliers or pseudocolor plots.

☒ A numerical value for number of cells or percentage (with statistics) is provided.

## Methodology

| | |
|---|---|
| Sample preparation | Semi-confluent MCF10A or U2OS cells were treated with 10uM EdU for 2 hrs prior to harvesting. Click-iT Plus EdU Alexa Fluor 488 Flow Cytometry Assay Kit (Invitrogen C10632) with the addition of FxCycle PI/RNase Staining Solution (Invitrogen F10797) was used per manufacturer's protocol and flow cytometry of a minimum of 10000 cells per condition was performed on LSRFortessa with subsequent analysis with Flow Jo version 10.8.1 (Flow Jo, BD). |
| Instrument | BD LSRFortessa |
| Software | Flow Jo version 10.8.1 |
| Cell population abundance | 10000 cells analyzed for each condition (no events except dead cells excluded) |
| Gating strategy | See above |

☐ Tick this box to confirm that a figure exemplifying the gating strategy is provided in the Supplementary Information.
