# Supplementary figures and images for: APOBEC3B regulates R-loops and promotes transcription-associated mutagenesis in cancer
Source: Nat Genet. 2023 Sep 21;55(10):1721–34. doi: 10.1038/s41588-023-01504-w (PMC10562255; doi:10.1038/s41588-023-01504-w)

Figure 1

c

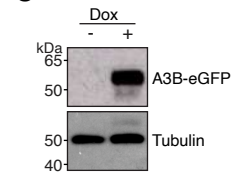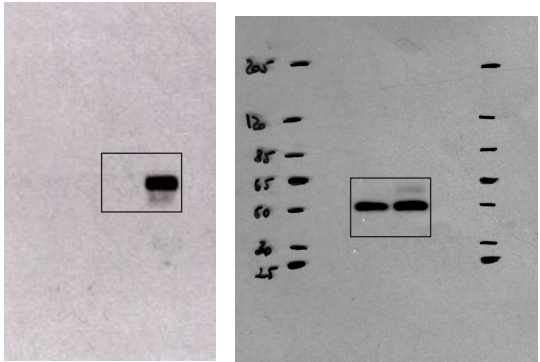

d

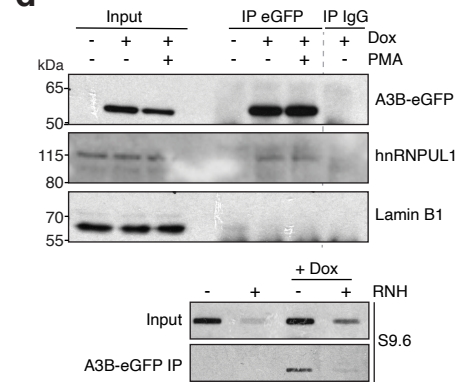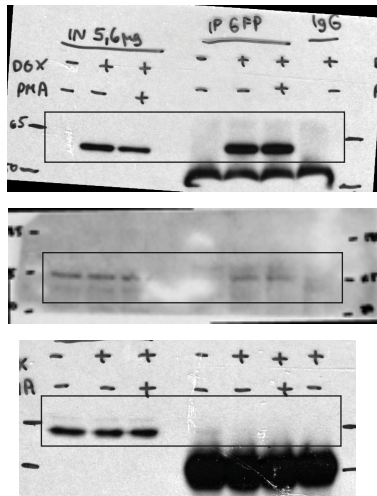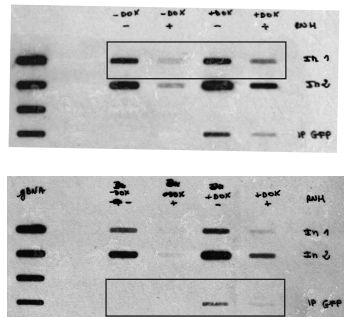

e

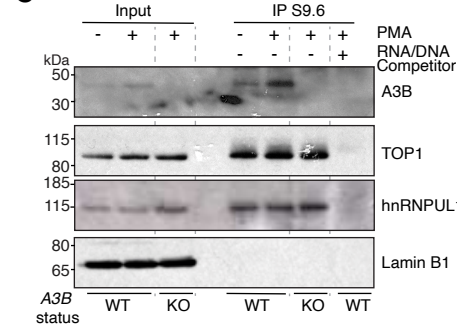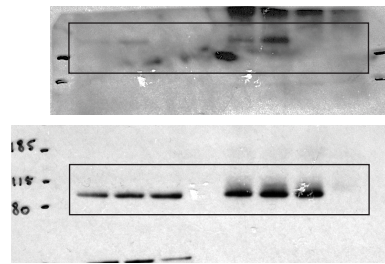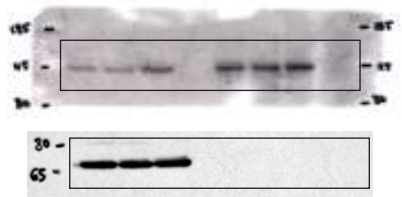

Supplement: Supplementary file 5 — Unprocessed immunoblots with relevant regions marked by boxes. [file 41588_2023_1504_MOESM5_ESM.pdf]

Figure 2

c

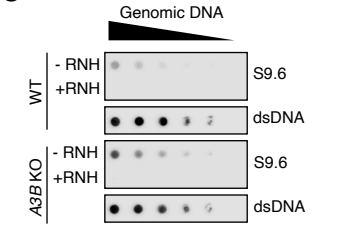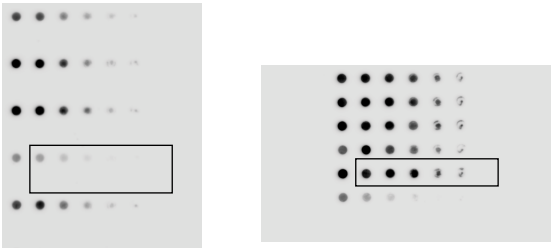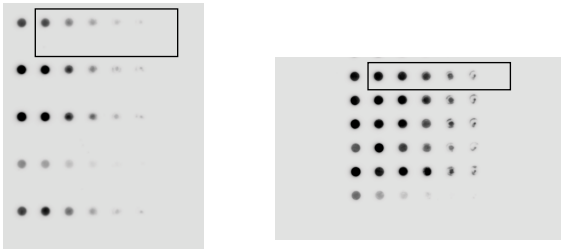

g

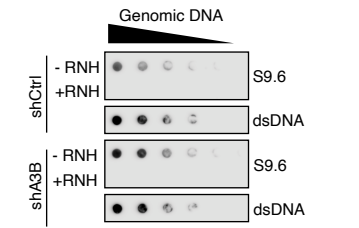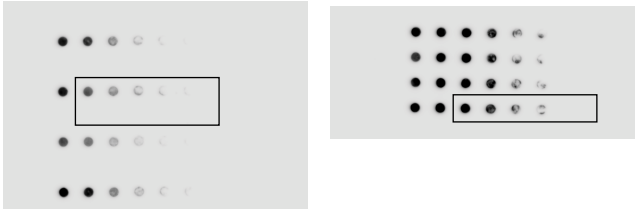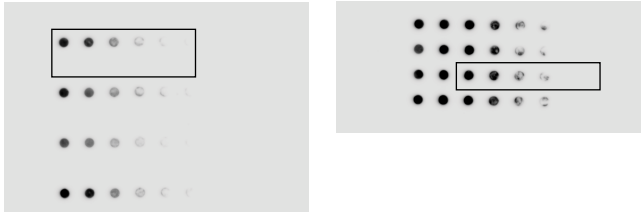

Supplement: Supplementary file 6 — Unprocessed dot blots with relevant regions marked by boxes. [file 41588_2023_1504_MOESM6_ESM.pdf]

Figure 3

i

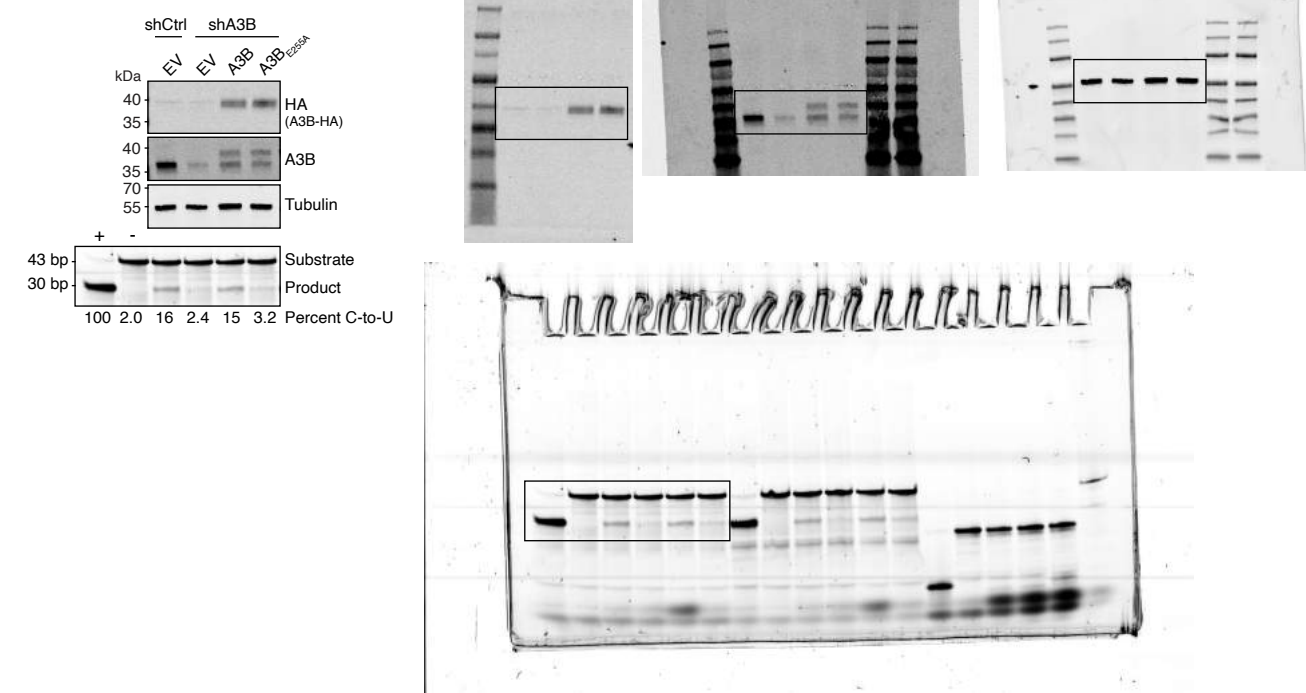

j

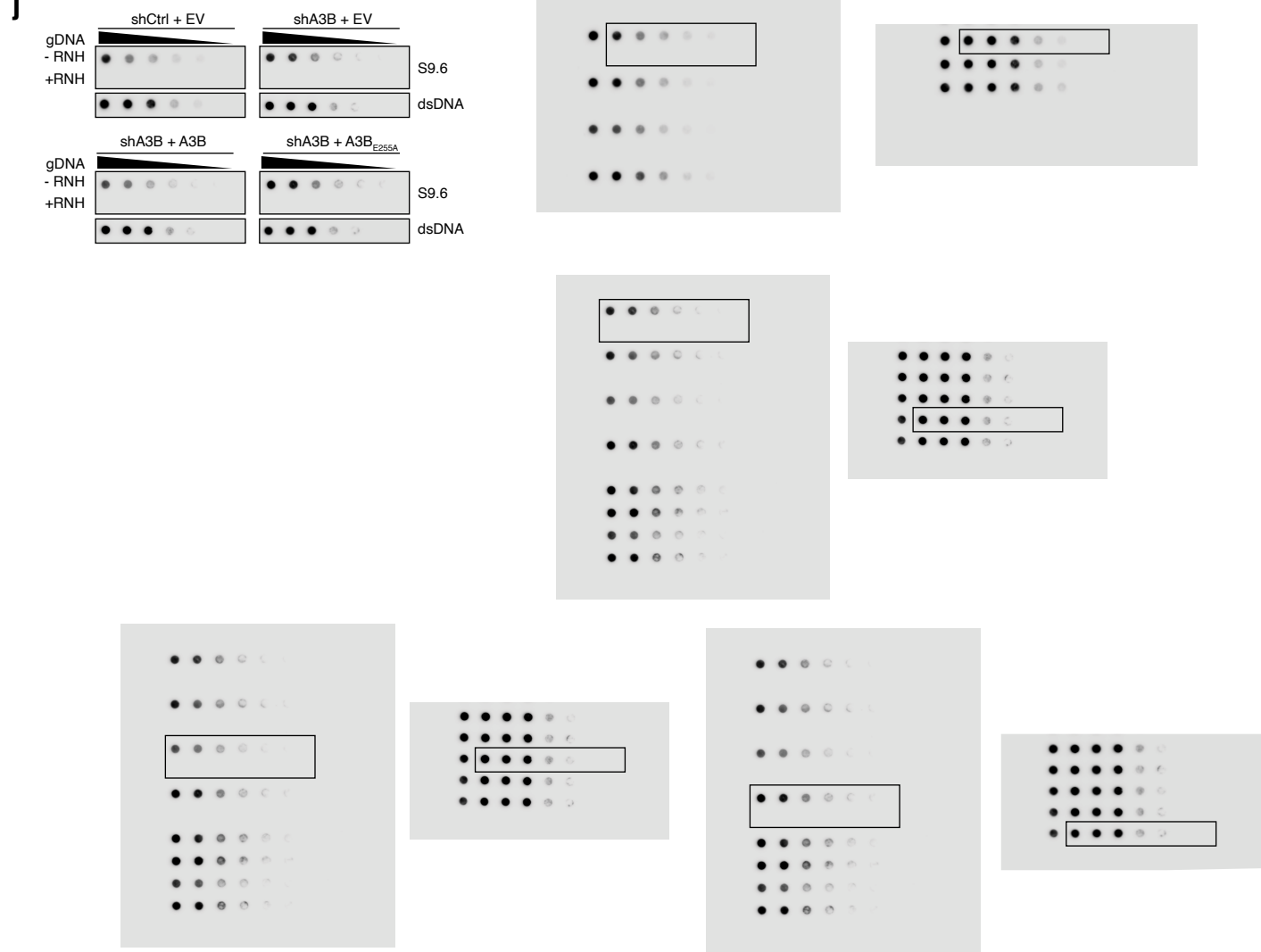

Supplement: Supplementary file 7 — Unprocessed immunoblots and dot blots with relevant regions marked by boxes. [file 41588_2023_1504_MOESM7_ESM.pdf]

Figure 4

c

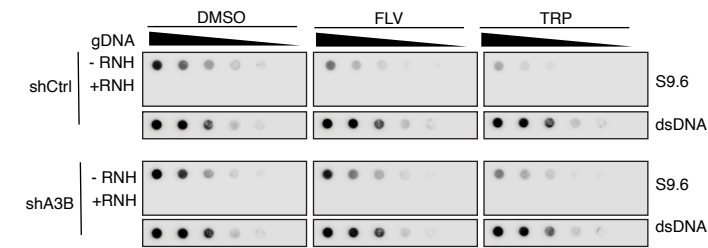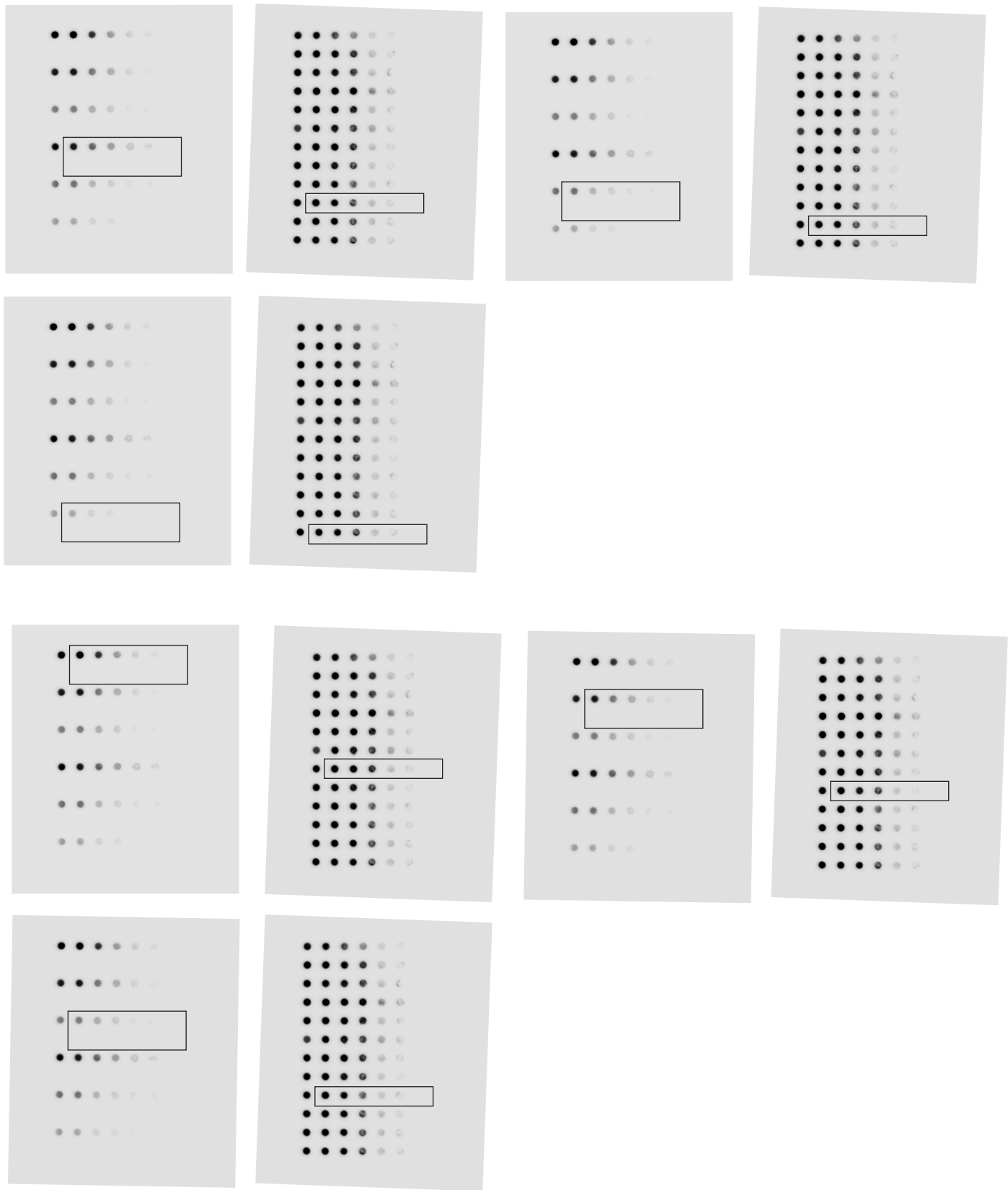

Supplement: Supplementary file 8 — Unprocessed dot blots with relevant regions marked by boxes. [file 41588_2023_1504_MOESM8_ESM.pdf]

Figure 6

c

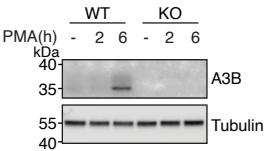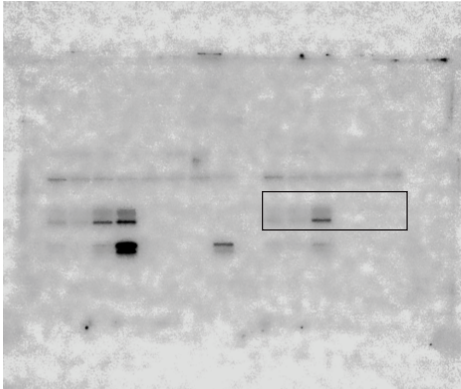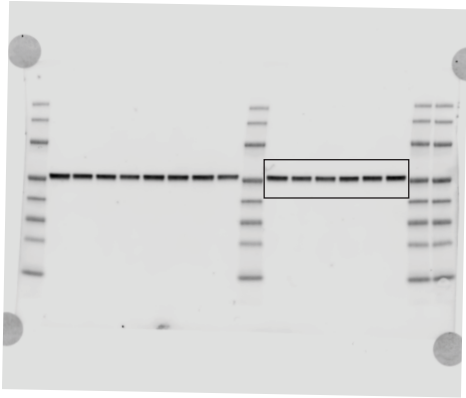

Supplement: Supplementary file 9 — Unprocessed immunoblots with relevant regions marked by boxes. [file 41588_2023_1504_MOESM9_ESM.pdf]

Figure 7

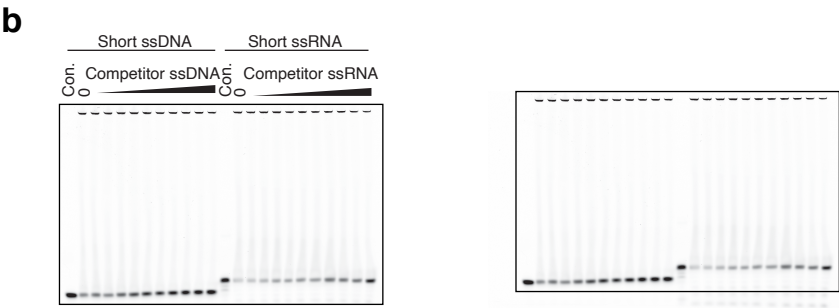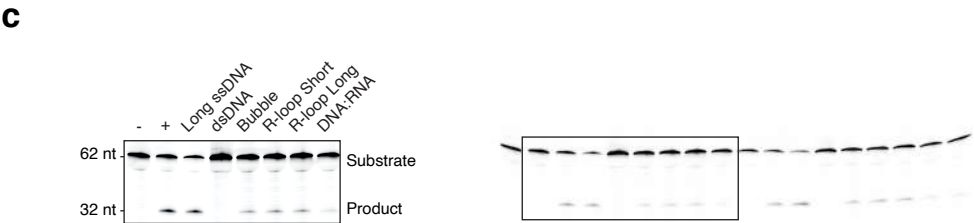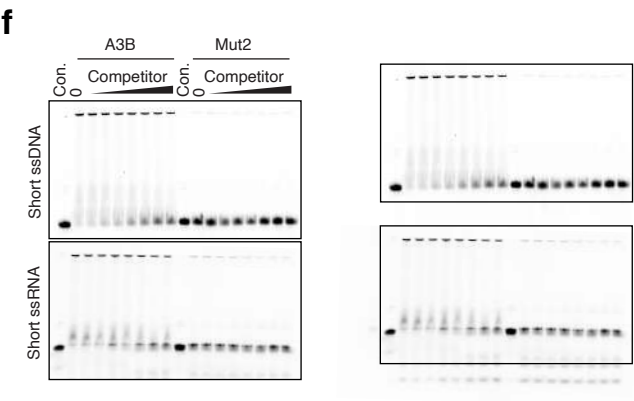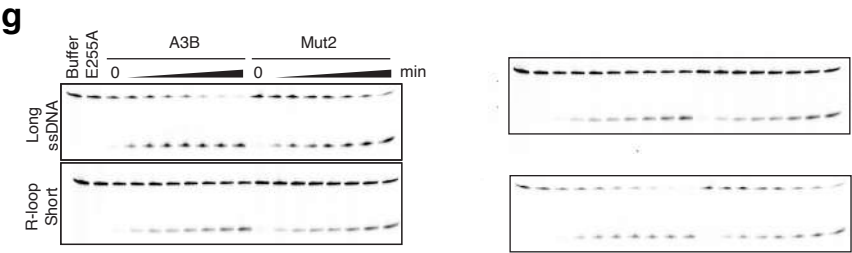

Supplement: Supplementary file 10 — Unprocessed gel images with relevant regions marked by boxes. [file 41588_2023_1504_MOESM10_ESM.pdf]

**Figure S1**

**b**

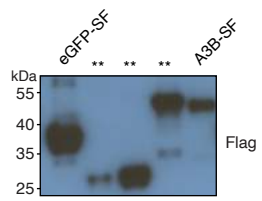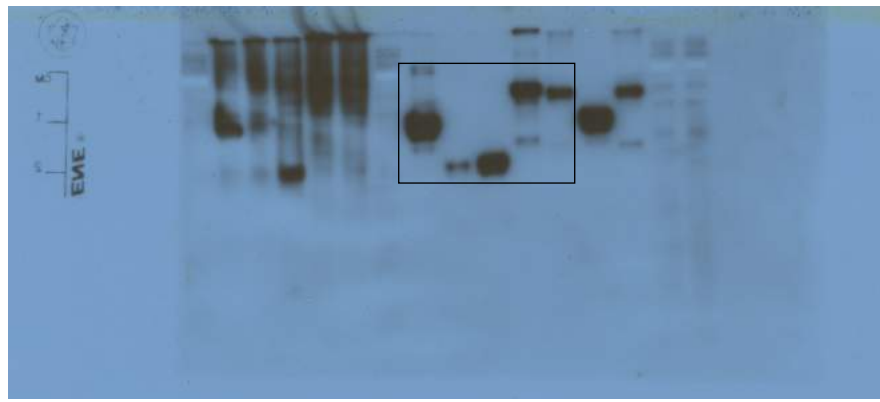

**c**

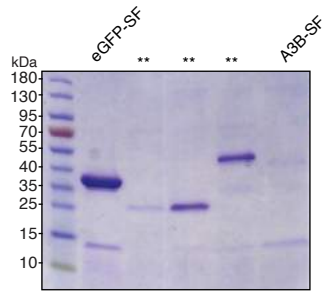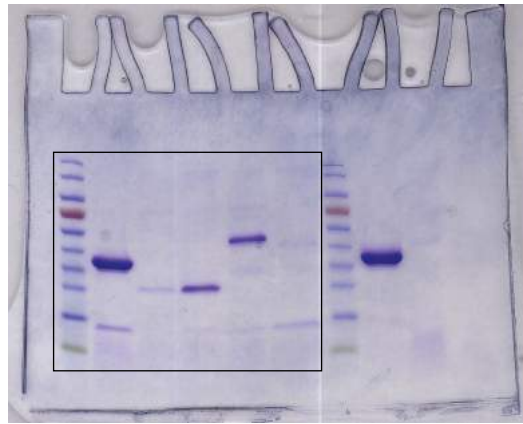

**d**

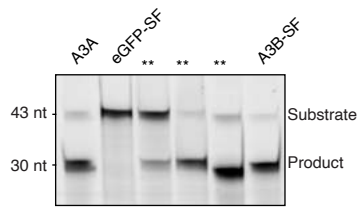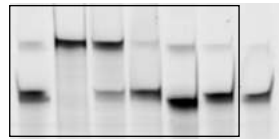

**Figure S1****e**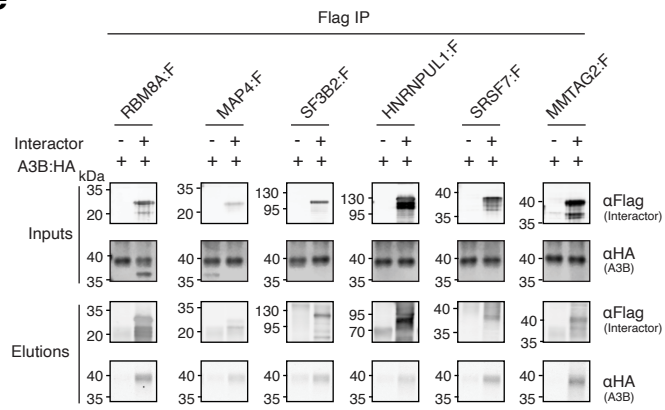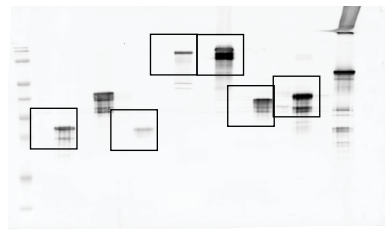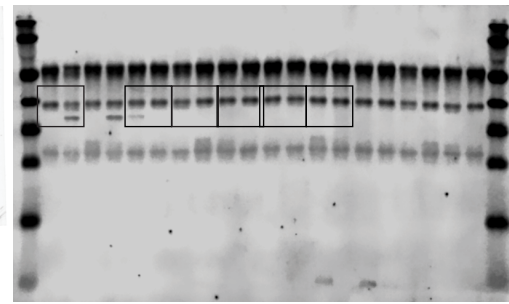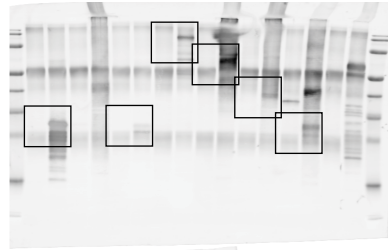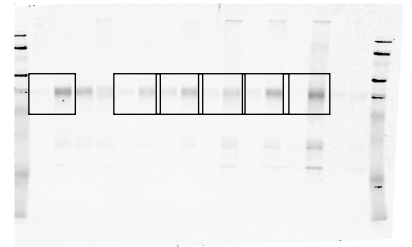**f**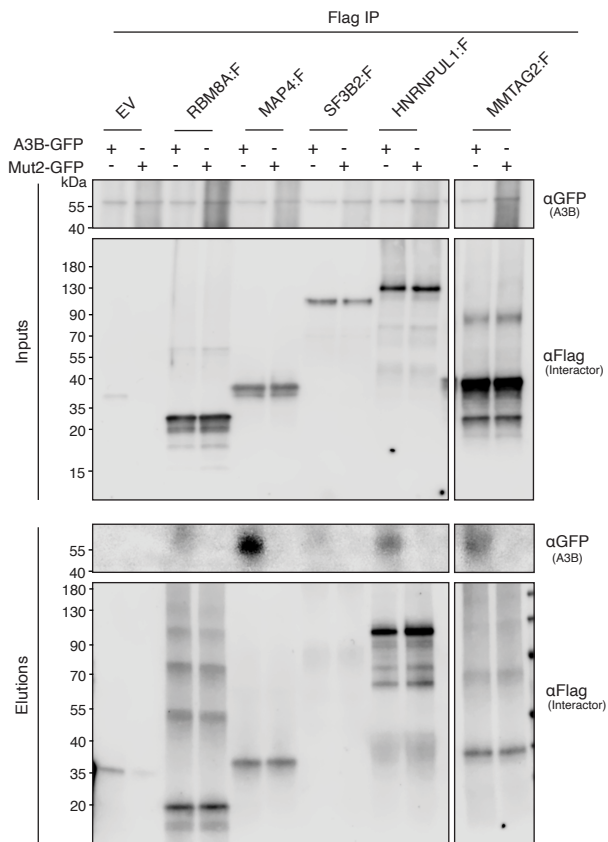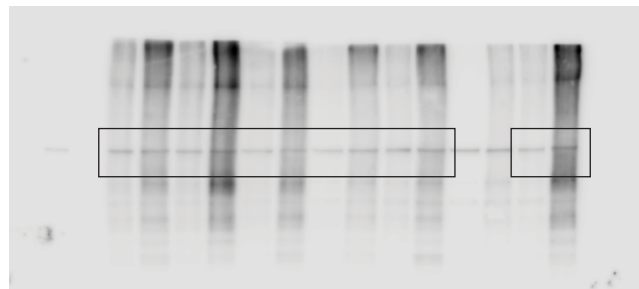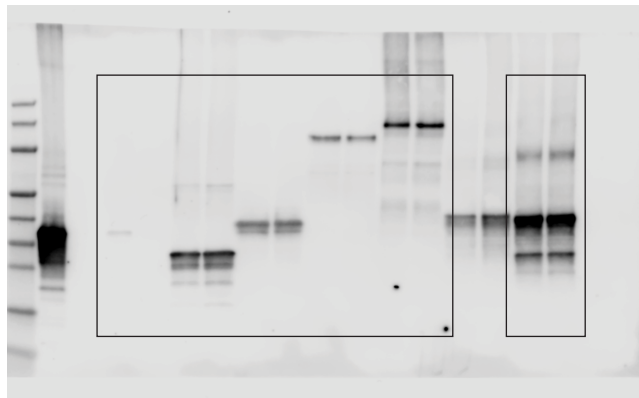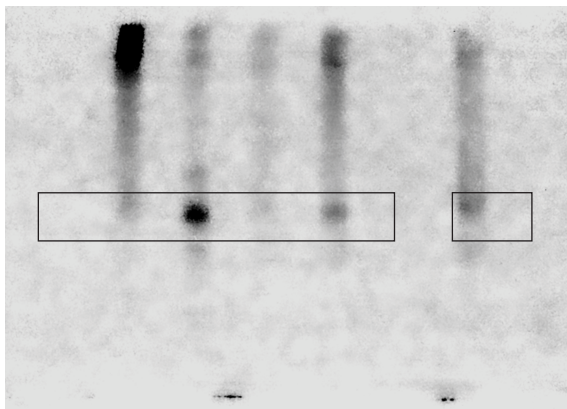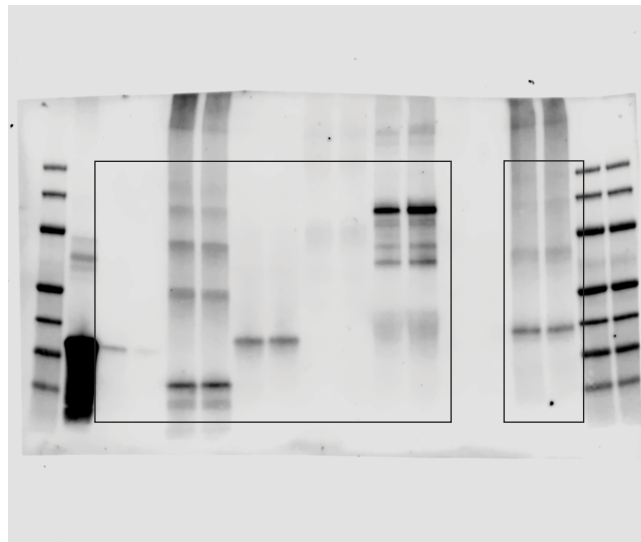

Supplement: Supplementary file 11 — Unprocessed gel and immunoblot images with relevant regions marked by boxes. [file 41588_2023_1504_MOESM11_ESM.pdf]

**Figure S2**

**b**

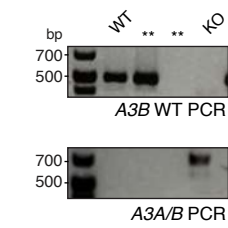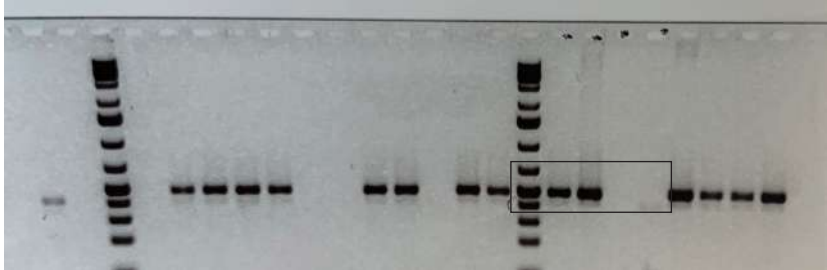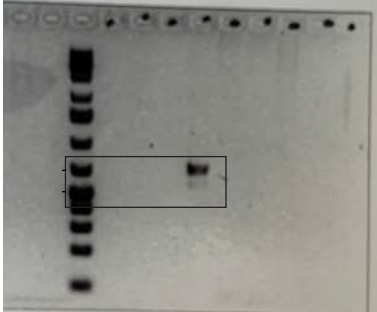

**c**

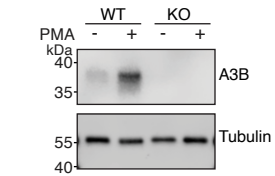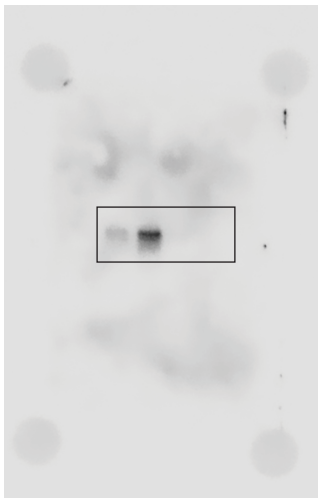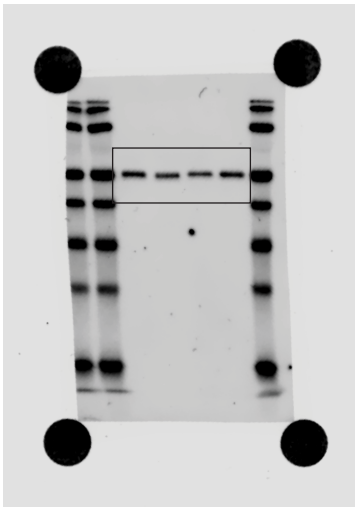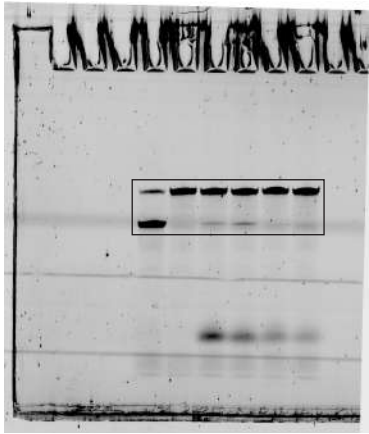

**d**

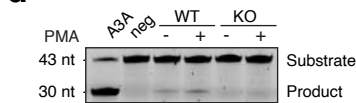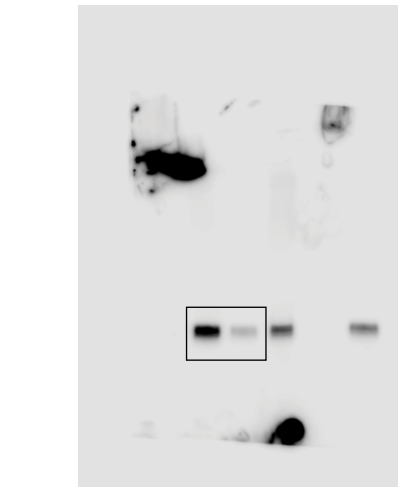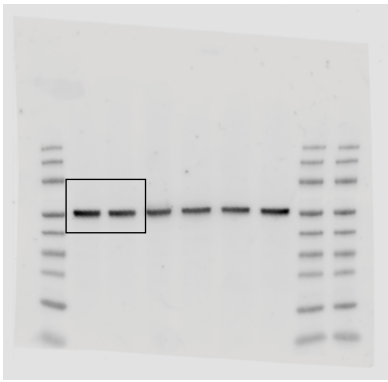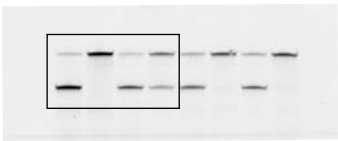

**f**

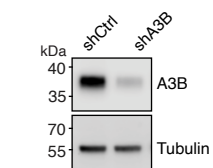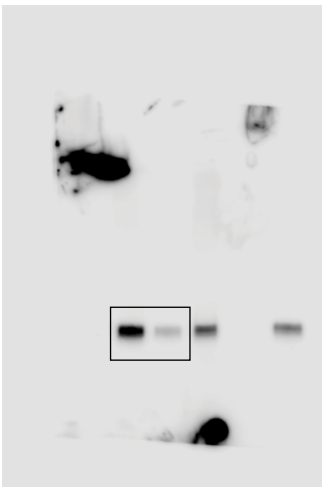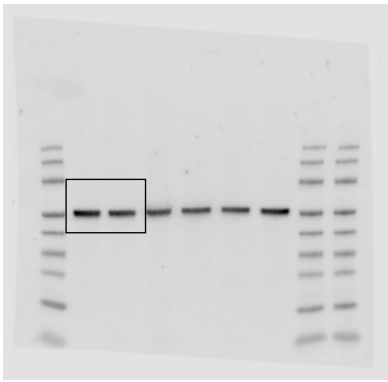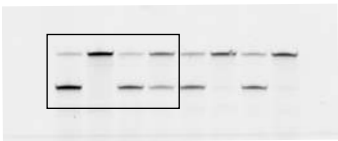

**g**

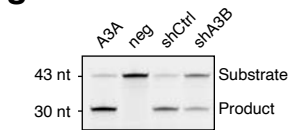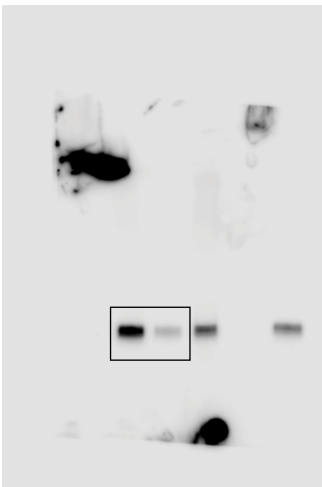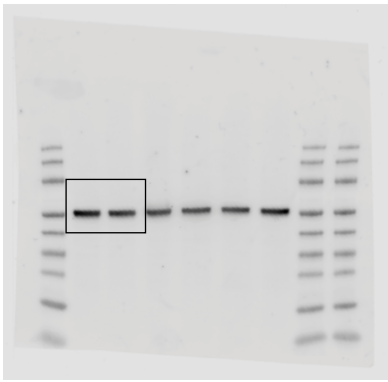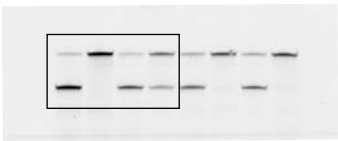

Figure S2

m

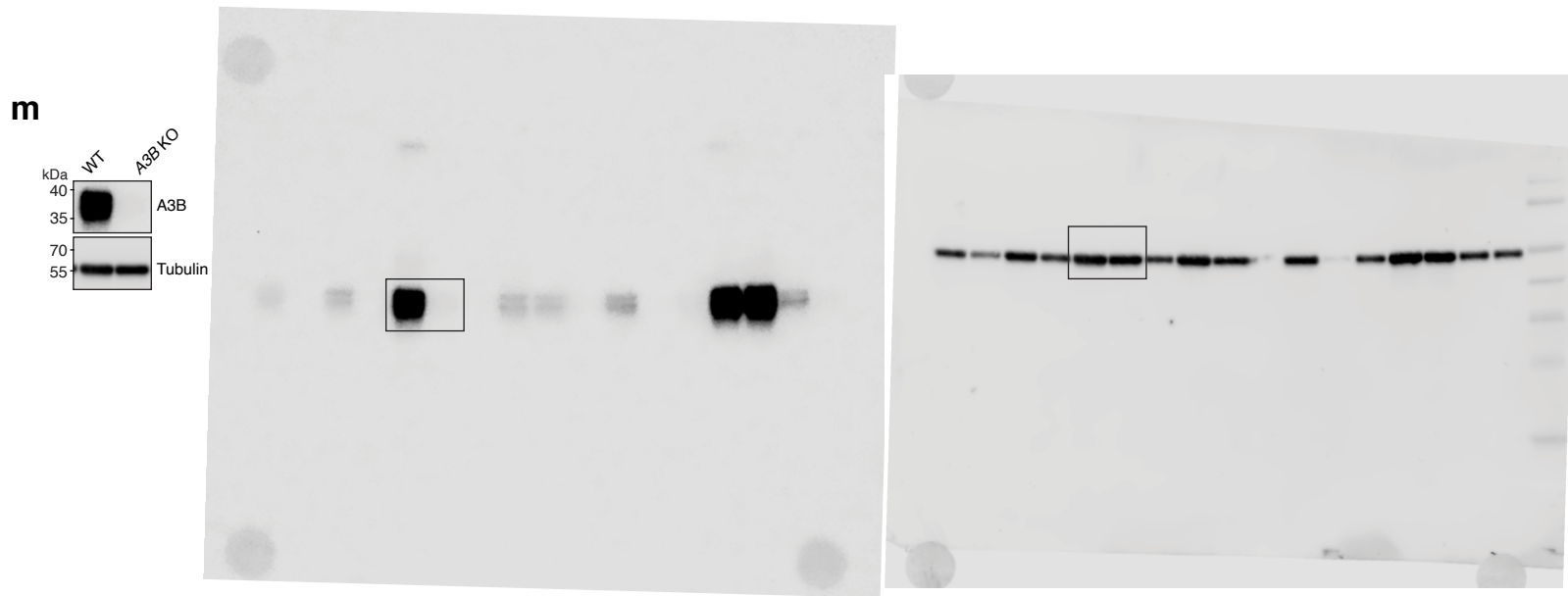

n

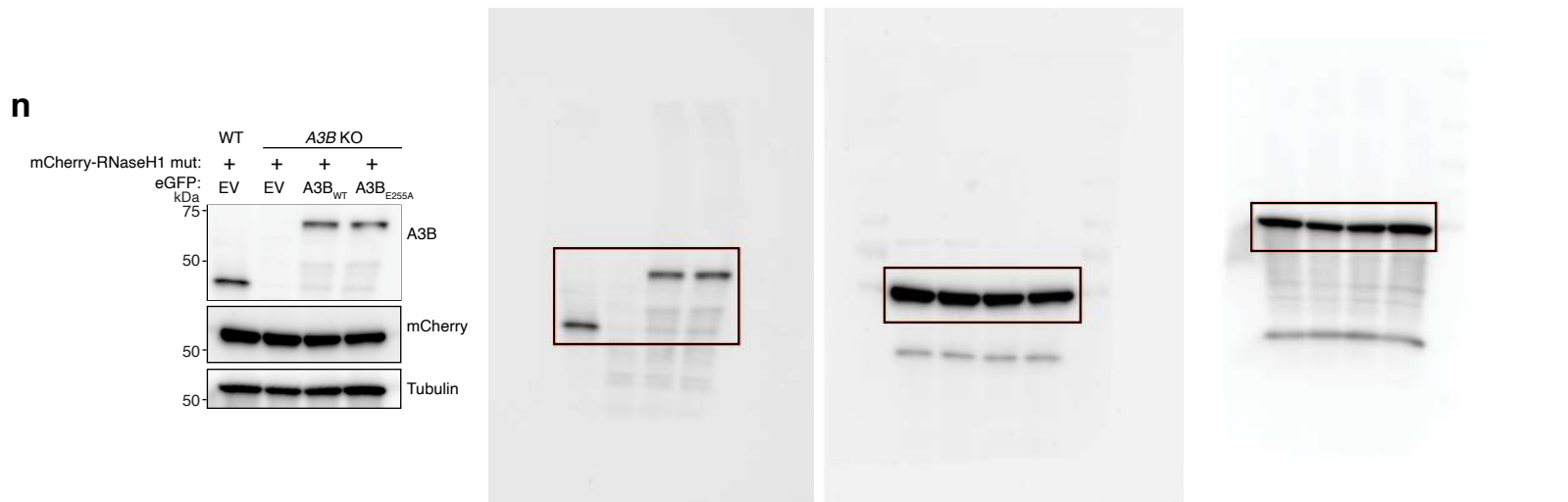

Supplement: Supplementary file 12 — Unprocessed gel and immunoblot images with relevant regions marked by boxes. [file 41588_2023_1504_MOESM12_ESM.pdf]

Figure S3

e

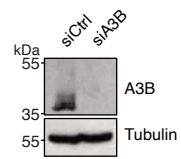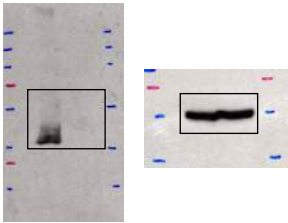

f

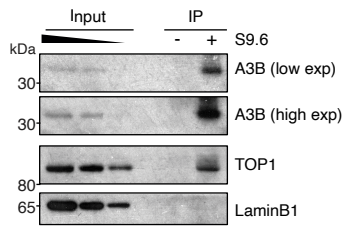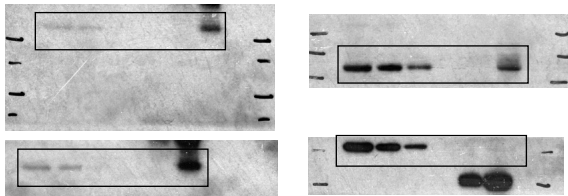

Supplement: Supplementary file 13 — Unprocessed gel and immunoblot images with relevant regions marked by boxes. [file 41588_2023_1504_MOESM13_ESM.pdf]

Figure S5

a

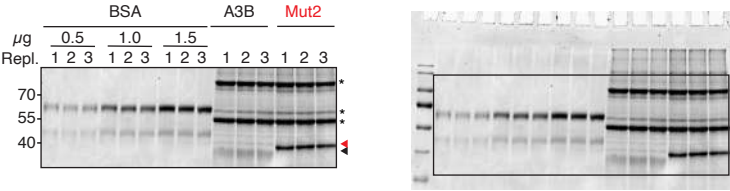

b

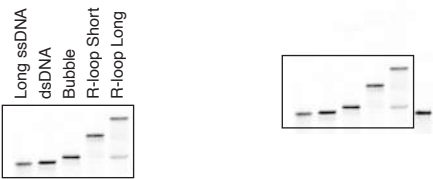

c

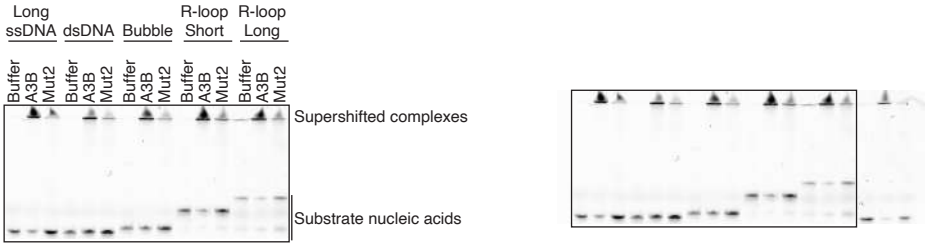

d

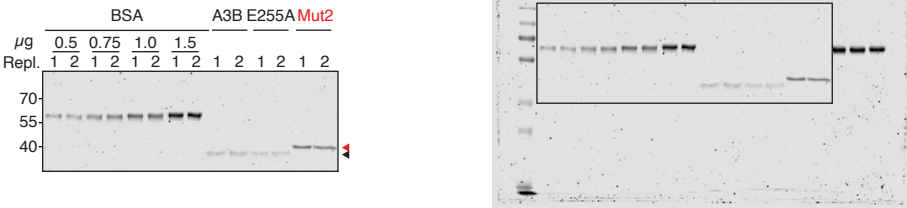

Figure S5

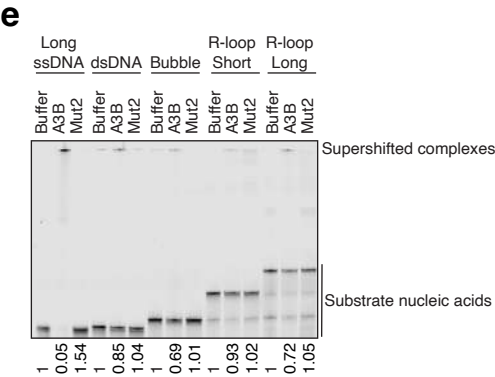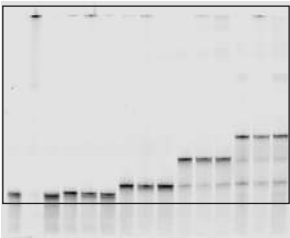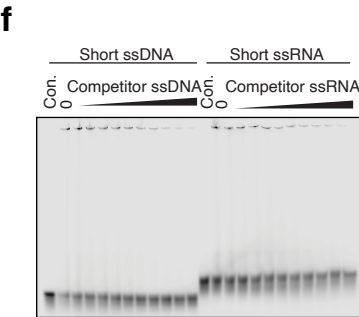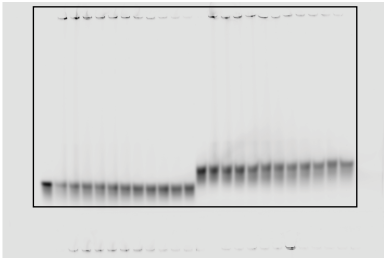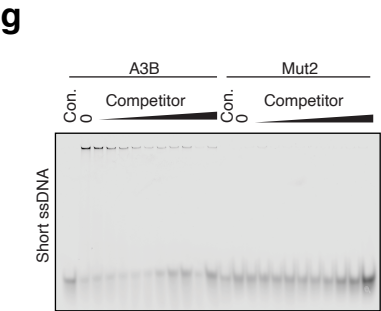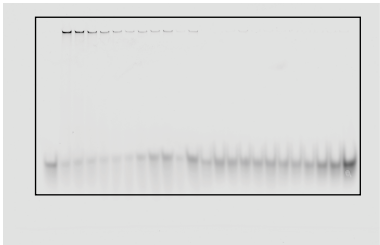

Supplement: Supplementary file 14 — Unprocessed gel images with relevant regions marked by boxes. [file 41588_2023_1504_MOESM14_ESM.pdf]
